# JAK2/IDH-mutant–driven myeloproliferative neoplasm is sensitive to combined targeted inhibition

**DOI:** 10.1172/JCI94516

**Published:** 2018-01-22

**Authors:** Anna Sophia McKenney, Allison N. Lau, Amritha Varshini Hanasoge Somasundara, Barbara Spitzer, Andrew M. Intlekofer, Jihae Ahn, Kaitlyn Shank, Franck T. Rapaport, Minal A. Patel, Efthymia Papalexi, Alan H. Shih, April Chiu, Elizaveta Freinkman, Esra A. Akbay, Mya Steadman, Raj Nagaraja, Katharine Yen, Julie Teruya-Feldstein, Kwok-Kin Wong, Raajit Rampal, Matthew G. Vander Heiden, Craig B. Thompson, Ross L. Levine

**Affiliations:** 1 Weill Cornell/Rockefeller/Sloan Kettering Tri-Institutional MD-PhD Program, New York, New York, USA.; 2 Gerstner Sloan Kettering Graduate School of Biomedical Sciences, and; 3 Human Oncology and Pathogenesis Program, Memorial Sloan Kettering Cancer Center, New York, New York, USA.; 4 Koch Institute for Integrative Cancer Research and Department of Biology, Massachusetts Institute of Technology, Cambridge, Massachusetts, USA.; 5 Lymphoma Service,; 6 Center for Hematologic Malignancies,; 7 Leukemia Service, Department of Medicine, and; 8 Department of Pathology, Memorial Sloan Kettering Cancer Center, New York, New York, USA.; 9 Whitehead Institute, Cambridge, Massachusetts, USA.; 10 Dana-Farber Cancer Institute, Boston, Massachusetts, USA.; 11 Agios Pharmaceuticals, Cambridge, Massachusetts, USA.; 12 Cancer Biology and Genetics Program, and; 13 Center for Epigenetics Research, Memorial Sloan Kettering Cancer Center, New York, New York, USA.

**Keywords:** Hematology, Oncology, Drug therapy, Leukemias

## Abstract

Patients with myeloproliferative neoplasms (MPNs) frequently progress to bone marrow failure or acute myeloid leukemia (AML), and mutations in epigenetic regulators such as the metabolic enzyme isocitrate dehydrogenase (IDH) are associated with poor outcomes. Here, we showed that combined expression of *Jak2*^V617F^ and mutant *IDH1*^R132H^ or *Idh2*^R140Q^ induces MPN progression, alters stem/progenitor cell function, and impairs differentiation in mice. *Jak2*^V617F^ *Idh2*^R140Q^–mutant MPNs were sensitive to small-molecule inhibition of IDH. Combined inhibition of JAK2 and IDH2 normalized the stem and progenitor cell compartments in the murine model and reduced disease burden to a greater extent than was seen with JAK inhibition alone. In addition, combined JAK2 and IDH2 inhibitor treatment also reversed aberrant gene expression in MPN stem cells and reversed the metabolite perturbations induced by concurrent *JAK2* and *IDH2* mutations. Combined JAK2 and IDH2 inhibitor therapy also showed cooperative efficacy in cells from MPN patients with both *JAK2*^mut^ and *IDH2*^mut^ mutations. Taken together, these data suggest that combined JAK and IDH inhibition may offer a therapeutic advantage in this high-risk MPN subtype.

## Introduction

Myeloproliferative neoplasms (MPNs) are clonal myeloid malignancies characterized by somatic mutations acquired in hematopoietic stem/progenitor cells, which drive the expansion of 1 or more myeloid lineages. Although patients with MPN can live with their disease for years, a subset of these patients progress to bone marrow failure or acute myeloid leukemia (AML), which are associated with poor clinical outcomes (1–4). Of note, patients with accelerated or transformed disease do not respond to conventional antileukemic therapies, including cytotoxic chemotherapy (1). As such, there is a need to identify biological features that drive MPN progression and to identify novel therapeutic targets in patients with high-risk or transformed MPN.

Gene discovery studies have underscored the pathogenic relevance of JAK/STAT activation in chronic MPNs, including mutations in *JAK2* (5), *MPL* (6), and *CALR* (7, 8). However, the genetic and epigenetic events that drive progression and induce adverse outcomes have not been fully delineated. Previous studies have identified recurrent somatic mutations in epigenetic regulators in patients whose disease has transformed from MPN to AML, including mutations in isocitrate dehydrogenase 1 (*IDH1*), *IDH2*, and *TET2* (9, 10). These studies are underscored by candidate gene studies in patients with chronic MPN, which have shown that mutations in epigenetic regulators are associated with poor overall survival (11, 12). Of note, patients with concurrent *JAK2* and *IDH1/2* mutations have shorter leukemia-free survival (11), suggesting that comutations in *JAK2* and in *IDH1/2* can promote MPN progression and transformation.

There is a need to elucidate how mutations in epigenetic regulators cooperate with JAK/STAT pathway-activating mutant alleles to promote transformation, with the hope that these insights will lead to the development of novel therapeutic approaches for this high-risk MPN subtype. Previous studies have shown that *Jak2^V617F^*-mutant disease is initiated and propagated in the long-term hematopoietic stem cell (LT-HSC) (13), and coexpression of the *Jak2^V617F^* allele with loss of *Tet2* manifests a more severe MPN phenotype (14). Furthermore, we and others have shown that the *Flt3^ITD^* allele can cooperate with *IDH2* gain of function or with loss of *Tet2* function to induce AML and that the resultant AML is initiated and maintained by the multipotent progenitor (MPP) cell population (15, 16). The neomorphic ability of mutant IDH to produce 2-hydroxyglutarate (2HG) (17, 18), a compound with epigenetic effects (19) on myeloid phenotype (20), underscores the importance of understanding the role of IDH in these diseases.

Models of combined *IDH1/2* and *JAK2* mutations provide a unique opportunity to explore the efficacy of combined targeted therapy against 2 gain-of-function disease alleles in the same leukemic clone. Previous studies have shown that JAK2 inhibitors can ameliorate symptoms and reduce spleen size in chronic MPN (21), and JAK inhibitor monotherapy can induce transient responses in patients with post-MPN AML (22). Preclinical studies with IDH inhibitors have been shown to inhibit mutant IDH1/2 function in vitro and in vivo and to induce responses in preclinical models, at least in part through induction of differentiation (23–25). IDH2 inhibitors are currently in clinical trials for patients with de novo AML, and early-phase clinical data have shown that these agents demonstrate efficacy with modest toxicity (26). We therefore sought to establish and characterize murine models of accelerated-phase MPN with concomitant JAK/STAT activation and IDH mutations to identify the disease mechanism and test novel mechanism-based combination therapies.

## Results

### JAK2^V617F^ and neomorphic IDH1/IDH2 mutations cooperate in vivo to drive progressive MPN.

**Figure 1:**
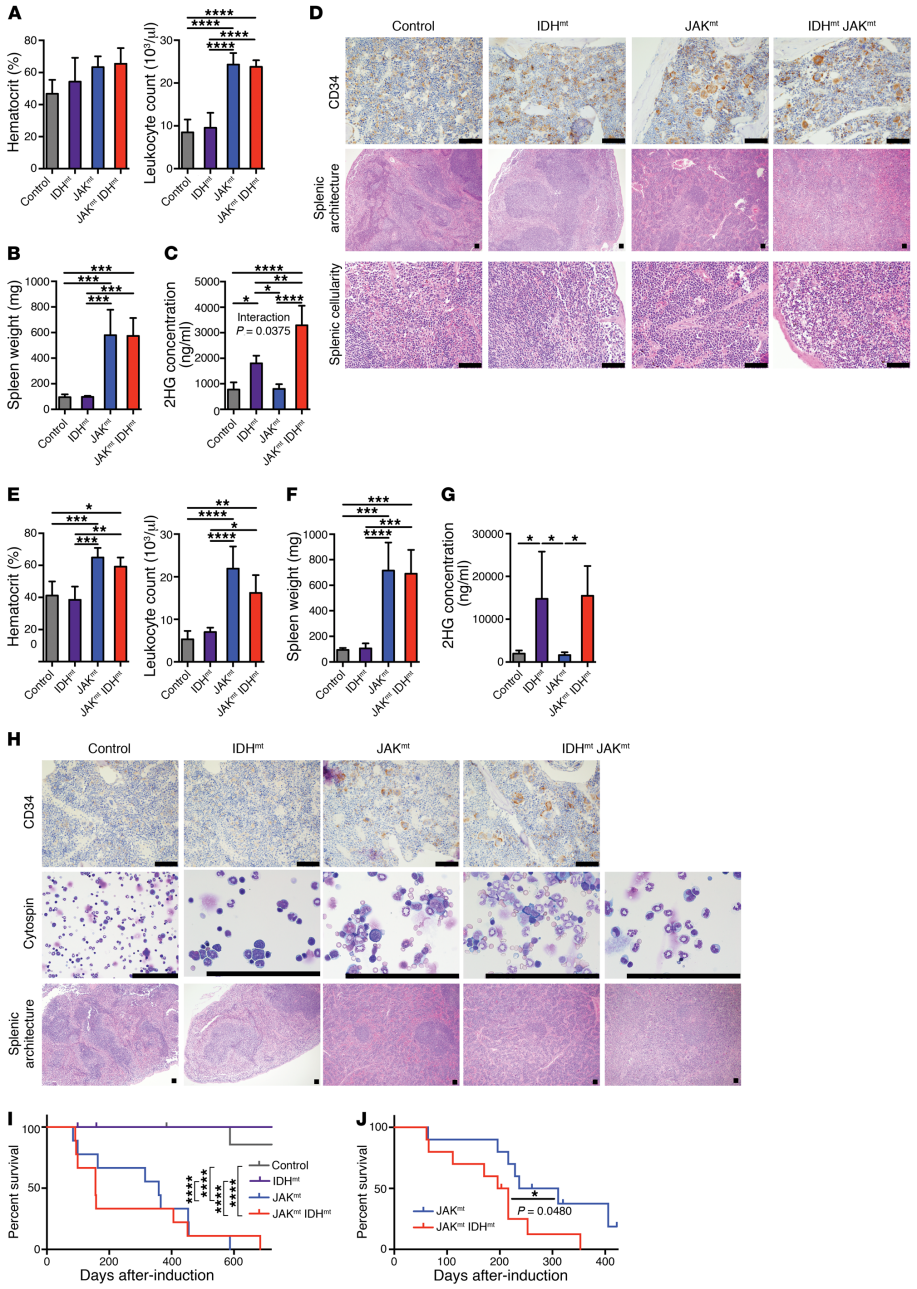
Combined *Jak2/IDH*-mutant mice have lethal MPN with preleukemic features. (**A**) Hematocrit levels and leukocyte counts in peripheral blood, (**B**) spleen weights, (**C**) 2HG levels in plasma, and (**D**) representative histology images for CD34 immunohistochemical stains of bone marrow and H&E stains of splenic tissue from primary *IDH1*^R132H^ *Jak2*^V617F^ Mx1-Cre mice sacrificed at approximately 6 months of age (*n* = 5/group). (**E**) Hematocrit levels and leukocyte counts in peripheral blood, (**F**) spleen weights, (**G**) 2HG levels in plasma, and (**H**) representative histology images for CD34 immunohistochemical stains of bone marrow, Wright-Giemsa stains of bone marrow cytospins, and H&E stains of splenic tissue from primary *Idh2*^R140Q^ *Jak2*^V617F^ Mx1-Cre mice sacrificed at approximately 6 months of age (*n* = 4–5/group). (**I)** Kaplan-Meier survival curve for primary *IDH1*^R132H^ *Jak2*^V617F^ Mx1-Cre mice following recombination. (**J)** Kaplan-Meier survival curve for secondary-transplant mice following injection of *IDH1*^R132H^ *Jak2*^V617F^ Mx1-Cre bone marrow. Scale bars: 200 μm. Multiple comparisons were performed using an ordinary 1-way ANOVA with Tukey’s correction for post-hoc comparisons and multiplicity-corrected *P* values. Comparisons of survival were performed using the log-rank (Mantel-Cox) test. Statistical interaction calculated for influence of Jak2 mutation status and IDH1 mutation status combined using 2-way ANOVA. *P < 0.05, **P < 0.01, ***P < 0.001, and ****P < 0.0001 by 2-way ANOVA. JAK^mt^, JAK-mutant; IDH^mt^, IDH-mutant; JAK^mt^/IDH^mt^, JAK/IDH-mutant.

In order to assess whether *IDH* and *JAK2* mutations cooperate to transform hematopoietic stem/progenitor cells, we crossed mice with conditional *IDH1*^R132H^ or *Idh2*^R140Q^ alleles (encoding human *IDH1* and murine *Idh2* mutations, respectively) with mice with a previously described *Jak2*^V617F^ (13) allele, and then used the inducible *Mx1-Cre* (27) allele to induce expression of these alleles in hematopoietic cells. Expression of *IDH1*^R132H^ or *Idh2*^R140Q^ in concert with *Jak2*^V617F^ resulted in a fully penetrant, lethal disease. In timed sacrifices at approximately 6 months of age, mice with combined mutations of *IDH1*^R132H^ or *Idh2*^R140Q^ and *Jak2*^V617F^ showed polycythemia (mean hematocrit levels of 59% and 65%, respectively), leukocytosis (mean leukocyte counts of 23.78 K/μl and 16.22 K/μl, respectively; 1, A and E), and splenomegaly (spleen weights of 574.4 mg and 690.7 mg, respectively; Figure 1, B and F), which was similar to what we observed in the *Jak2*-mutant controls. *IDH1*^R132H^ and *Idh2*^R140Q^ mice had elevated serum levels of 2HG (Figure 1, C and G), and 2HG levels were higher in mice with concurrent *IDH1*^R132H^ and *Jak2*^V617F^ mutations compared with mice with the *IDH1*^R132H^ mutation alone (*P* = 0.0024), suggesting that *Jak2* and *IDH1* mutations interact to promote higher 2HG levels (interaction *P* = 0.0375; Figure 1C) through either increased 2HG production or higher IDH-mutant cell burden. Expression of mutant *IDH1* or *Idh2* in concert with *Jak2*^V617F^ resulted in disruption of the splenic architecture beyond that observed in *Jak2*^V617F^ mice, including the expansion of blast-like cells with open chromatin and large nucleoli in JAK2/IDH-mutant mice, which was not observed in *Jak2*^V617F^ mice. We found that JAK2/IDH-mutant megakaryocytes had increased expression of CD34 by IHC compared with platelet progenitors in *Jak2*^V617F^ mice, which was consistent with impaired megakaryocytic differentiation (Figure 1, D and H). Expression of *IDH1*^R132H^ in concert with *Jak2*^V617F^ resulted in an overall survival similar to that of mice with expression of *Jak2*^V617F^ alone (median survival, 156 and 359 days, respectively; NS), but lower than that of *IDH1*^R132H^-mutant and WT mice (median survival was undefined in an 800-day period; *P* < 0.0001; Figure 1I). Mice transplanted with *Jak2*^V617F^*/IDH1*^R132H^-mutant cells showed significantly reduced survival compared with recipients transplanted with *Jak2*-mutant cells (median survival of 206 and 274 days, respectively; *P* = 0.0480; Figure 1J). Of note, previous studies showed that expression of *Idh2*^R140Q^ was indicative of survival rates that were similar to those observed with our *IDH1*^R132H^ models (28). These data indicate that concurrent JAK2 and IDH1/2 mutations cooperate to drive a lethal, transplantable MPN with impaired differentiation in vivo.

### JAK2/IDH-mutant MPN initiates and propagates disease from the LT-HSC compartment.

**Figure 2:**
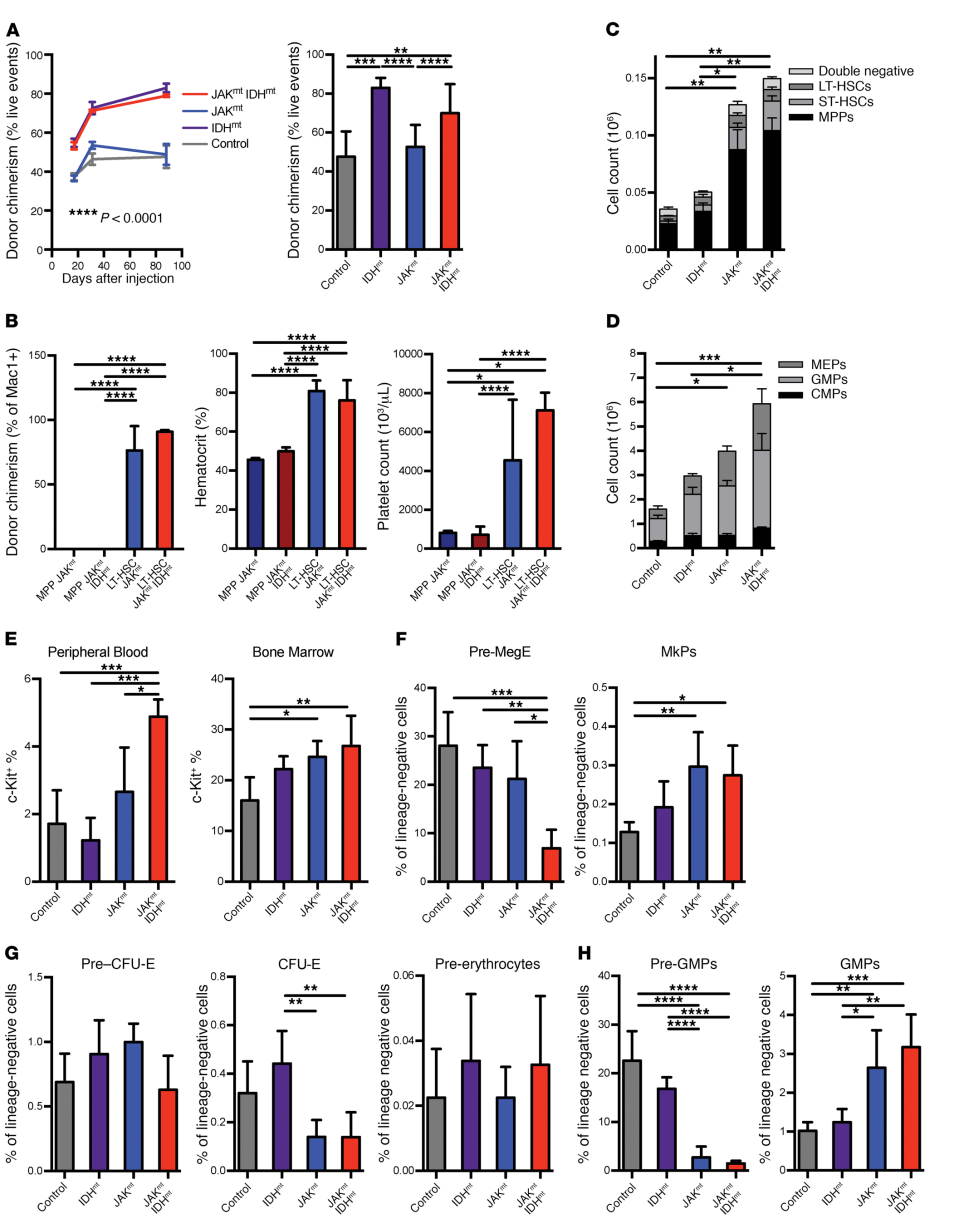
Combined mutant mice have expanded pathological stem and progenitor cell populations. (**A**) Peripheral blood donor chimerism of competitive transplants with *Idh2*^R140Q^ *Jak2*^V617F^ bone marrow over time (*n* = 5/group) and at 12 weeks (*n* = 5–50/group). (**B**) Peripheral blood chimerism, hematocrit levels, and platelet counts in recipients of bone marrow sorted for MPP or LT-HSC LSK populations, 15 weeks after injection (*n* = 5/group). (**C**) Total number of LSK cells and (**D**) total number of myeloprogenitor cells in bone marrow from primary *Idh2*^R140Q^ *Jak2*^V617F^ mice and controls according to stem cell/progenitor compartment as measured by FACS (*n* = 4–5/group). (**E**) Stem cell populations as measured by FACS in peripheral blood and bone marrow from primary *Idh2^R140Q^ Jak2^V617F^* mice, expressed as a percentage of lineage-negative cells. (**F**) MkP cell populations, (**G**) erythrocytic progenitor cell populations, and (**H**) granulocytic progenitor cell populations as measured by FACS in bone marrow from primary *Idh2^R140Q^ Jak2^V617F^* mice, expressed as a proportion of lineage-negative cells (*n* = 4–5/group). Multiple comparisons were performed using an ordinary 1-way ANOVA with Tukey’s correction for post-hoc comparisons and multiplicity-corrected *P* values. *P < 0.05, **P < 0.01, ***P < 0.001, and ****P < 0.0001.

Given that the MPN driven by concurrent JAK2/IDH mutations had more efficient disease transplantation, we next sought to define which stem/progenitor cell populations could propagate the disease. In competitive transplants comparing combined mutant marrow with WT CD45.1^+^ marrow, we found that *Idh2*^R140Q^-mutant bone marrow cells were able to out-compete WT cells, and the resultant increase in self-renewal induced by mutant *Idh2* was maintained in cells with concurrent *Idh2*^R140Q^ and *Jak2*^V617F^ mutations (Figure 2A). Transplant recipients had a phenotype similar to that of primary mice, including polycythemia and thrombocytosis. Notably, we observed greater leukocytosis in mice engrafted with *Idh2*^R140Q^ *Jak2*^V617F^ marrow compared with mice transplanted with *Jak2*^V617F^-mutant cells (Supplemental Figure 1A; supplemental material available online with this article; https://doi.org/10.1172/JCI94516DS1).

Because previous studies have suggested that hematopoietic malignancies induced by mutant IDH and *Jak2* propagate through cells derived from different stem cell compartments (13, 15, 16), we sought to explore the cell population that could propagate JAK2/IDH-mutant disease. *IDH1*^R132H^ and *Jak2*^V617F^ bone marrow cells were sorted into LSK (Lin^–^CD117/c-Kit^+^Sca1^+^), LT-HSC (LSK CD48^–^CD150^+^), and MPP (LSK CD48^+^CD150^–^) (Supplemental Figure 1B) populations and transplanted into congenic recipient mice. We assessed disease chimerism and peripheral blood counts in the recipient mice and found that mice transplanted with LT-HSCs, but not MPPs, had evidence of long-term engraftment and myeloproliferation (Figure 2B). LT-HSC–transplanted recipients developed a lethal MPN consistent with efficient propagation of the disease from the stem cell compartment (Supplemental Figure 1C). These data demonstrate that JAK2/IDH-mutant MPN is initiated and propagated in LT-HSCs, in contrast to IDH- and *Tet2*-mutant AML models, in which leukemic stem cell capacity is maintained in the MPP cell population.

We next assessed the relative number of stem/progenitor cells in JAK2/IDH-mutant mice and found that total LSK cell numbers had expanded, with an increase in all hematopoietic stem and progenitor cell compartments compared with WT and IDH single mutants (Figure 2C and Supplemental Figure 1, D and E). Concurrent JAK2/IDH mutations drove an increase in myeloid progenitors (MPs) (Lin^–^Sca^+^c-Kit^–^), including an increase in common myeloid progenitors (CMPs) (Lin^–^Sca^+^c-Kit^–^CD16/32^–^CD34^+^; Figure 2G, Supplemental Figure 1, F and G). We found that the proportion of myeloid (Mac1^+^B220^–^) cells that expressed c-Kit was increased in double mutants compared with IDH-only (*P* = 0.0002) and JAK2-only (*P* = 0.0150) single-mutant and nonmutant (*P* = 0.0008) mice (Figure 2E). To explore this further, we examined more granular aspects of the myeloid lineage. Pre-megakaryocyte (pre-MegE) populations (Lin^–^c-Kit^+^Sca1^–^CD41^–^CD16/32^–^CD150^+^CD105^–^) were significantly reduced in *Idh2*^R140Q^ *Jak2*^V617F^ combined mutants compared with *Jak2*^V617F^ mutants alone, while megakaryocyte progenitors (MkPs) (Lin^–^c-Kit^+^Sca1^–^CD150^+^CD41^+^) were expanded in *Jak2*^V617F^ mice, regardless of IDH status (Figure 2F). With respect to erythroid progenitors, concurrent JAK2 and IDH2 mutations resulted in a reduction in CFU erythrocytes (CFU-E) (Lin^–^c-Kit^+^Sca1^–^CD41^–^CD16/32^–^CD150^–^CD105^+^Ter119^–^) and an increase in pre-erythrocytes (Lin^–^c-Kit^+^Sca1^–^CD41^–^CD16/32^–^CD150^–^CD105^+^Ter119^+^) (Figure 2G). In contrast, pre–granulocyte-macrophage progenitors (pre-GMPs) (Lin^–^c-Kit^+^Sca1^–^CD41^–^CD16/32^–^CD150^–^CD105^–^) were reduced, while granulocyte-macrophage progenitors (GMPs) (Lin^–^c-Kit^+^Sca1^–^CD41^–^CD16/32^+^CD150^–^) were proportionally expanded (Figure 2H). These data indicate that combined mutant MPN shows perturbations in stem, progenitor, and precursor cell populations, with an increase in more primitive stem/progenitor cells and a decrease in mature megakaryocyte/erythroid populations.

### Combined JAK2/IDH2 inhibition shows increased efficacy in JAK2/IDH2-mutant MPN.

**Figure 3:**
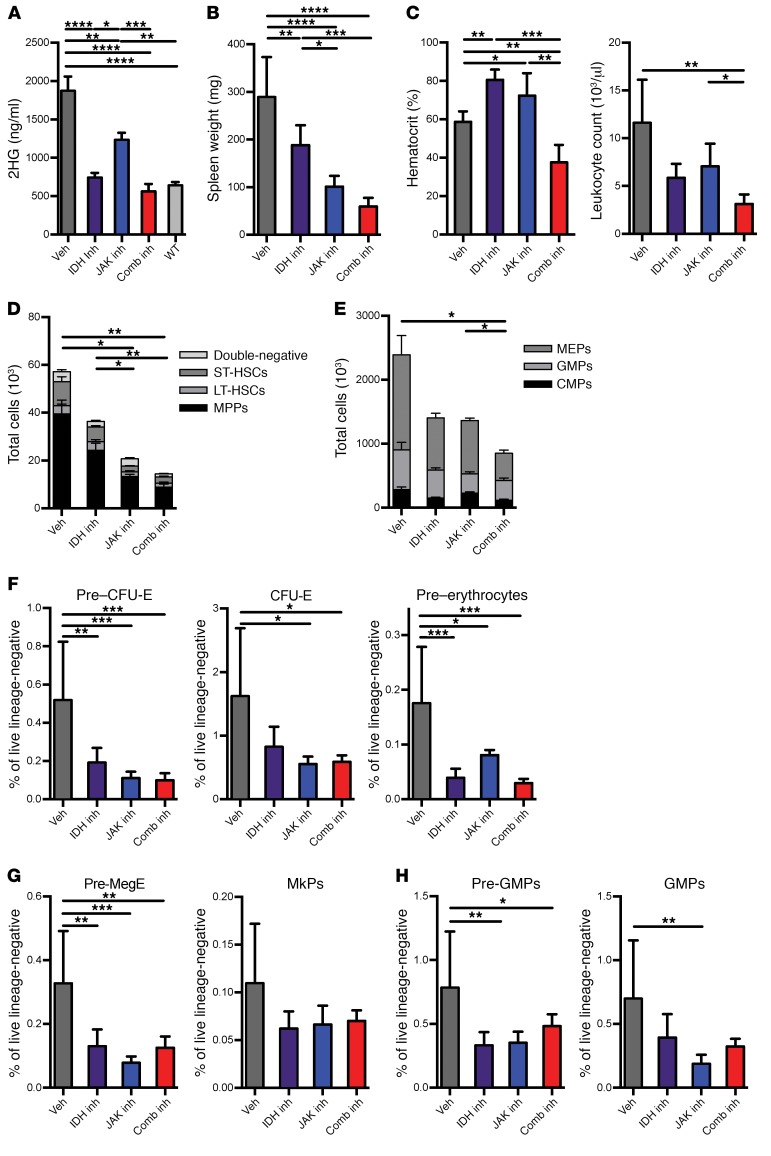
Treatment of combined mutant mice results in the resolution of disease phenotype. (**A**–**H**) Disease phenotype in transplant recipients of *Idh2*^R140Q^ *Jak2*^V617F^ bone marrow treated with targeted inhibitors at sacrifice after 4 weeks of treatment (*n* = 7–10/group). (**A**) 2HG levels in plasma, (**B**) spleen weights, (**C**) hematocrit levels and leukocyte counts, (**D**) total LSK cells classified by compartment, (**E**) total myeloprogenitors classified by compartment, (**F**) erythrocytic progenitors, (**G**) MkPs, and (**H**) granulocytic progenitors as measured by FACS. Multiple comparisons were performed using an ordinary 1-way ANOVA with Tukey’s correction for post-hoc comparisons and multiplicity-corrected *P* values. Paired *t* tests were used to determine 2-tailed significance for comparison of results in mice before and after treatment. *P < 0.05, **P < 0.01, ***P < 0.001, and ****P < 0.0001. Comb, combined; inh, inhibitor; Veh, vehicle.

Given that MPN patients with concurrent JAK2 and IDH mutations are at high risk for disease progression and have adverse clinical outcomes, we tested whether JAK2 inhibition, IDH2 inhibition, or combined JAK2/IDH2 inhibition would show efficacy in treating *Jak2*/*Idh2*-mutant MPN. We engrafted mice with equal ratios of CD45.2 *Jak2*/*Idh2*-mutant cells and CD45.1 WT cells, and after the recipient mice developed MPN, they were treated with vehicle, the IDH2 inhibitor AG221, and/or the JAK kinase inhibitor ruxolitinib (INC18424). We observed no evidence of synergistic or additive toxicity with combination therapy, and ruxolitinib therapy did not increase AG221 levels. Serum 2HG levels in mice engrafted with *Idh2*^R140Q^ *Jak2*^V617F^–mutant cells (mean, 1,874 ng/ml) were reduced with oral AG221 therapy at doses between 40 and 100 mg/kg given as monotherapy (mean, 744.4; *P* < 0.0001) or in combination with ruxolitinib (mean, 562.6 ng/ml; *P* < 0.0001), consistent with target inhibition (Figure 3A). Interestingly, ruxolitinib monotherapy also modestly reduced serum 2HG levels (mean, 1,233 ng/ml; *P* = 0.0016; Figure 3A). Splenomegaly in diseased mice (mean vehicle-treated spleen weight, 289.1 mg) was reduced by AG221 monotherapy (188.3 mg; *P* = 0.0040) or ruxolitinib monotherapy (101.3 mg; *P* < 0.0001), but splenomegaly resolved completely with combined therapy (59.53 mg; *P* < 0.0001; Figure 3B). Combined therapy with AG221 and ruxolitinib also normalized polycythemia (hematocrit levels of 58.7% vs. 37.61%; *P* = 0.0028) and leukocytosis (leukocyte counts of 11.62 K/μl vs. 3.111 K/μl; *P* = 0.0069) to an extent beyond that observed with either agent alone (Figure 3C). Total LSK cell numbers in the bone marrow of double-mutant mice (mean for treatment with vehicle, 57.19 × 10^3^ cells) was reduced by AG221 monotherapy (36.43 × 10^3^ cells; NS), ruxolitinib monotherapy (20.73 × 10^3^ cells; *P* = 0.0266), or combined treatment (14.42 × 10^3^ cells; *P* = 0.0090); a similar reduction in stem/progenitor cells was observed in LT-HSCs, short-term hematopoietic stem cell (ST-HSC ) (LSK CD48^+^CD150^–^), and MPPs (Figure 3D and Supplemental Figure 1H). Combined treatment reduced total MP numbers in the bone marrow of double-mutant mice (mean for treatment with vehicle, 2.46 × 10^6^ vs. 0.8863 × 10^6^) to an extent greater than that seen with either agent alone, and this reduction was observed in all measured subpopulations including CMPs, GMPs, and megakaryocyte-erythroid progenitors (MEPs) (Figure 3E and Supplemental Figure 1I).

We also found that the expansion of erythrocyte progenitors was reduced with either ruxolitinib or AG221 monotherapy or with combined therapy. These included pre–CFU-E (mean for treatment with vehicle, 0.5189% vs. AG221, 0.1913% [*P* = 0.0051] vs. ruxolitinib, 0.1103% [*P* = 0.0005] vs. combined therapy, 0.09978% [*P* = 0.0006]); CFU-E (mean for treatment with vehicle, 1.621 vs. AG 0.8251 [*P* = NS] vs. ruxolitinib, 0.5553 [*P* = 0.0105] vs. combination therapy, 0.5868 [*P* = 0.0180]); and pre-erythrocytes (mean for treatment with vehicle, 0.1757 vs. AG221, 0.03906 [*P* = 0.0005] vs. ruxolitinib, 0.08063 [*P* = 0.0149] vs. combination therapy, 0.02943 [*P* = 0.0003]) (Figure 3F). Treatment with either monotherapy or combined therapy reduced pre-MegE populations (mean for treatment with vehicle, 0.1757 vs. AG221, 0.03906 [*P* = 0.0005] vs. ruxolitinib, 0.08063 [*P* = 0.0149] vs. combined therapy, 0.02943 [*P* = 0.0003]), with a trend toward a reduction in the expanded MkP populations in all treatment groups (Figure 3G). Similarly, combined therapy or monotherapy reduced pre-GMP and GMP populations (Figure 3H).

### IDH2 inhibition or combined JAK2/IDH2 inhibition reduces disease burden in vivo.

**Figure 4:**
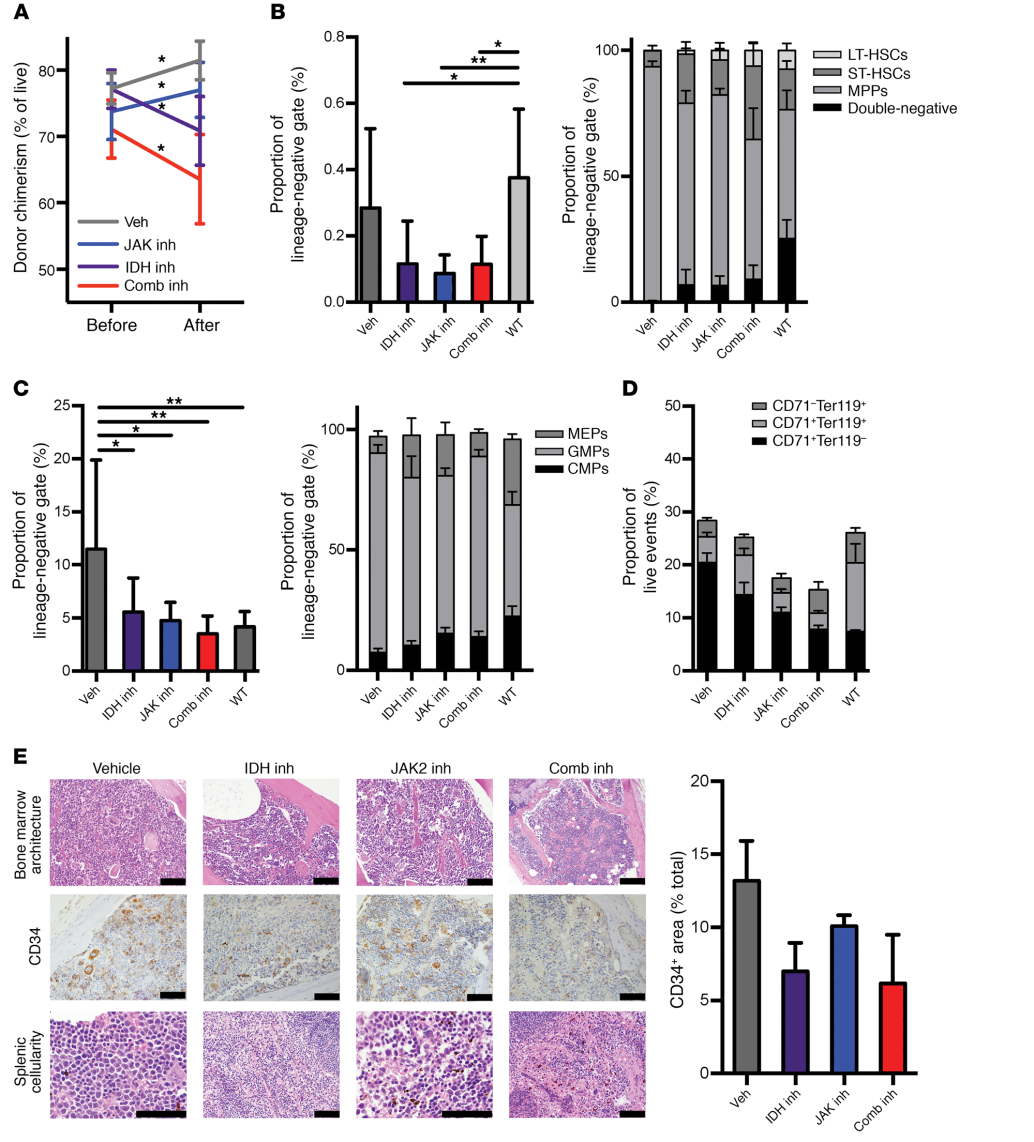
Treatment of combined mutant mice results in reduced disease chimerism. (**A**) Paired evaluation of donor chimerism in peripheral blood of *Idh2*^R140Q^ *Jak2*^V617F^ bone marrow recipients before and after treatment with targeted inhibitors for 4 weeks (*n* = 7/group). (**B**–**D**) Stem cell phenotype within the donor (CD45.2^+^) compartment of *Idh2*^R140Q^ *Jak2*^V617F^ bone marrow recipients treated with targeted inhibitors (*n* = 10/group). (**B**) Total LSK compartments expressed as a proportion of the total lineage-negative gate and as proportions of LSK subcompartments. (**C**) Total myeloid progenitors expressed as a proportion of the total lineage-negative gate and as proportions of LSK subcompartments. (**D**) Erythroid progenitors expressed as proportions of early-, middle-, and late-maturity cells. (**E**) Representative images of bone marrow morphology with dilatations, CD34 immunohistochemical staining in the bone marrow with digital quantification of staining (graph), and splenic cell/blast morphology in *Idh2*^R140Q^ *Jak2*^V617F^ bone marrow recipients treated with targeted inhibitors. Scale bars: 200 μm. Multiple comparisons were performed using an ordinary 1-way ANOVA with Tukey’s correction for post-hoc comparisons and multiplicity-corrected *P* values. Paired *t* tests were used to determine 2-tailed significance for comparison of results in mice before and after treatment. *P < 0.05, and **P < 0.01.

Since JAK2 inhibitors do not substantively reduce allele burden in preclinical models of *JAK2*-mutant disease (29) or in the clinical context (21, 30), we next assessed whether IDH2 inhibition, alone or in combination with JAK2 inhibition, could reduce disease burden in vivo. We assessed the impact of AG221 therapy or AG221-ruxolitinib combined therapy on the proportion of *Idh2*^R140Q^ *Jak2*^V617F^ MPN cells (CD45.2^+^) in recipient mice. In comparing allele burden in the peripheral blood from individual mice before and after treatment, we observed that mice treated with either AG221 or combined JAK2/IDH2 inhibition showed significant reductions in mutant donor chimerism (mean change before and after treatment for AG221, –6.291%, *P* = 0.0411, and for combination, –7.547%, *P* = 0.0225). In contrast, mice treated with either ruxolitinib or vehicle showed a significant expansion of mutant-derived cell burden (mean change before and after treatment for ruxolitinib, +3.256%, *P* = 0.0122, and for vehicle, +4.236, *P* = 0.0374) (Figure 4A). Examination of only donor-derived (CD45.2^+^) cells revealed that monotherapy with either inhibitor or combined IDH2/JAK2 inhibitor therapy reduced the proportion of CD45.2^+^ LSK cells (Figure 4B), whereas the number of CD45.1^+^ WT stem/progenitor cells was not affected (data not shown), indicating a potent, selective effect of AG221 on mutant cells in vivo. Within the donor-derived LSK compartment, LSK LT-HSC, ST-HSC, and MPP subpopulations returned to WT levels (Figure 4B). Consistent with the effect on stem/progenitor cells, we found that IDH2 inhibitor monotherapy or combined IDH2/JAK2 inhibitor therapy reduced the proportion of CD45.2^+^ MP populations (Figure 4C) without affecting the number of CD45.1^+^ MPs (data not shown). Within the donor-derived MP compartment, subpopulations including CMPs, GMPs, and MEPs were also partially normalized with either monotherapy or combined therapy (Figure 4C). The proportion of CD45.2-mutant erythroid progenitor (CD71^+^Ter119^–^) cells was normalized with combination therapy, consistent with a potent suppression of mutant erythroid progenitors (Figure 4D).

Histologically, treatment with JAK2/IDH2 combination therapy normalized bone marrow and splenic morphology, with a reduction in the proportion of myeloid cells and the development of bone cortex to normal patterns of dilatation by histopathological analysis. Treatment with AG221, but not ruxolitinib, reduced the aberrant CD34 expression seen in megakaryocytes, whereas combined treatment eliminated the presence of these cells. We also observed that AG221 or AG221-ruxolitinib combination therapy eliminated the expansion of splenic blasts observed in untreated JAK2/IDH2-mutant MPN (Figure 4E).

### Combined JAK2/IDH2 inhibition normalizes aberrant transcription in JAK2/IDH2-mutant MPN.

**Figure 5:**
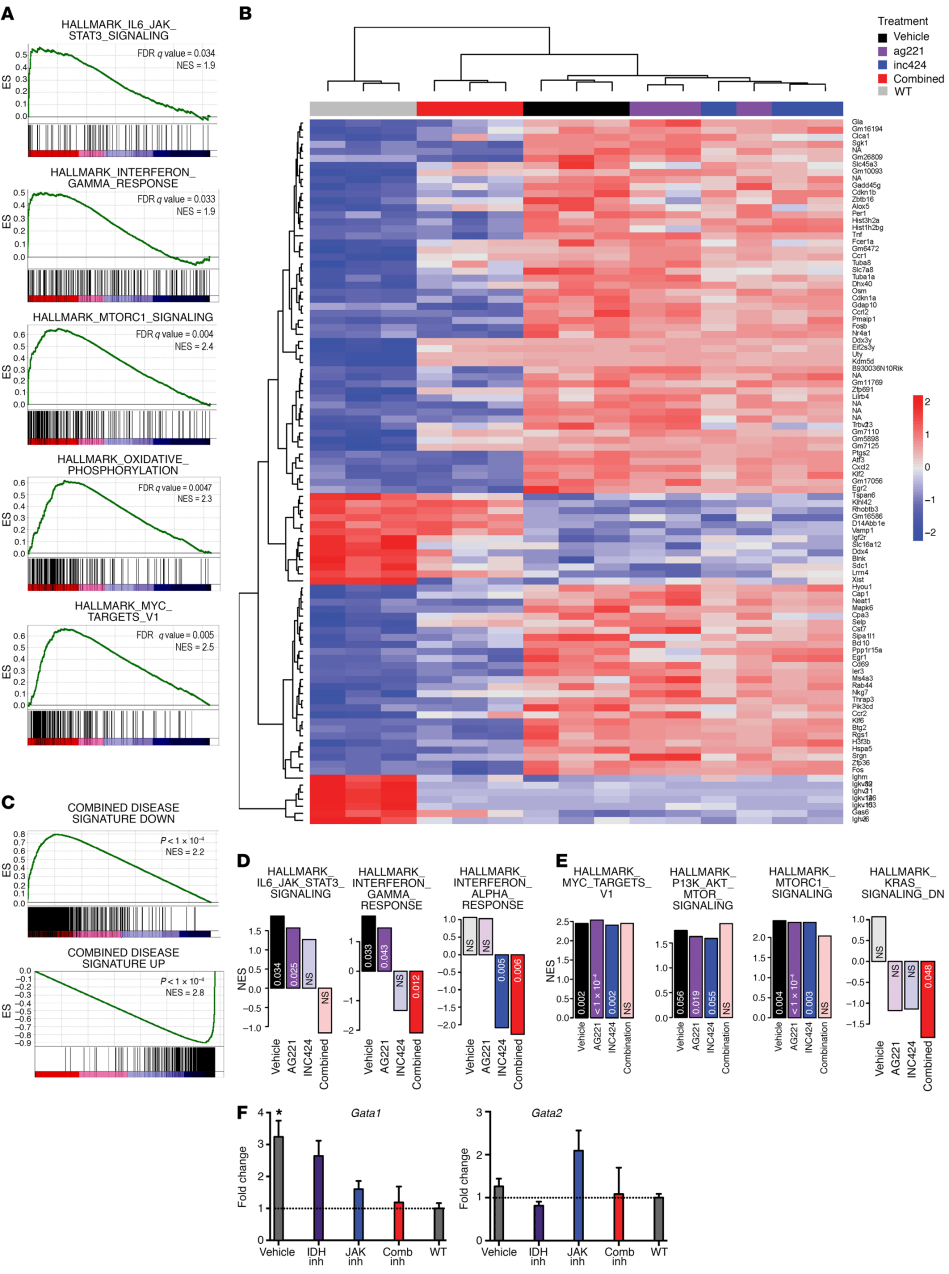
Expression in donor-derived (CD45.2^+^) LSK cells by RNA-seq defines the gene set of combined mutant disease, and treatment eradicates this expression profile. (**A**–**E**) RNA-seq analysis of donor derived (CD45.2^+^) LSK cells in *Idh2*^R140Q^ *Jak2*^V617F^ bone marrow recipients treated with targeted inhibitors (*n* = 3/group). (**A**) Significantly enriched Hallmark GSEA in comparative expression patterns comparing vehicle/diseased mice to WT. (**B**) Clustering of all treated mice depicting comparative phylogeny of samples and relative expression of the 100 most significantly differentially expressed genes. (**C**) Examination of genes differentially expressed between combined treatment mice and vehicle-treated mice using a gene set defined according to genes differentially expressed between WT and vehicle-treated mice. (**D** and **E**) Calculated normalized enrichment score (NES) values (*y* axis) and FDR (*x* axis) of curated Hallmark GSEA showing the level and significance of enrichment in each treatment group compared with WT examined for enrichment in curated Hallmark GSEA lists related to JAK/STAT signaling. Statistically significantly non-zero NES values are depicted in bright colors, while nonstatistically significant NES values are depicted in pastel colors. (**D**) Hallmark GSEA highlighting several pathways related to JAK/STAT signaling and (**E**) several oncogenic pathways. (**F**) Quantitative PCR for *Gata1* and *Gata2* expression performed on sorted MEPs from drug-treated mice. See Methods for details on the statistical methods used for the bioinformatics analysis in **A** and **C**. Multiple comparisons were performed using an ordinary 1-way ANOVA with Tukey’s correction for post-hoc comparisons and multiplicity-corrected *P* values. *P < 0.05.

Given the role of JAK2 as a transcriptional activator and the ability of mutant IDH to modulate the epigenetic state (19, 20), we assessed whether combined JAK2/IDH2 mutations affected gene expression in vivo and whether this was abrogated by combined IDH2/JAK2 inhibition. We harvested and sorted mutant CD45.2 LSK cells from recipient mice engrafted with JAK2/IDH2-mutant cells that were treated with vehicle, AG221, ruxolitinib, or combination therapy and compared their transcriptional output with each other and with WT cells through RNA sequencing (RNA-seq). *Idh2*^R140Q^ *Jak2*^V617F^–mutant LSK cells had a distinct gene expression profile compared with that of WT LSK cells. This gene expression profile demonstrated enrichment in MSigDB Hallmark gene sets related to JAK/STAT signaling including IL-6/JAK/STAT3 signaling (*q* = 0.034; NES = 1.9) and IFN-γ signaling (*q* = 0.033; NES = 1.9) gene sets (Figure 5A). We also observed enrichment in several gene expression sets related to metabolism, including mTOR (*q* = 0.005; NES = 2.4) and oxidative phosphorylation (*q* = 0.0047; NES = 2.3) (Figure 5A). Finally, the expression of specific oncogenic signatures such as cMYC (*q* = 0.004; NES = 2.5) was increased in JAK2/IDH2-mutant cells. Together, these data suggest that concurrent *Idh2*^R140Q^ and *Jak2*^V617F^ mutations result in transcriptional alterations.

Inhibitor treatment had an impact on aberrant gene expression in *Idh2*^R140Q^ *Jak2*^V617F^–mutant LSK cells. We observed a more dramatic effect on gene expression with combination JAK2/IDH2 inhibitor therapy than with either monotherapy, such that LSK cells derived from mice receiving combination therapy had clustering most similar to that seen among WT LSK cells in unsupervised analysis (data not shown) and in clustering based on differentially expressed genes between diseased and WT mice (Figure 5B). The perturbations of these genes in vehicle-treated mice compared with WT mice were consistent with previous data sets published on a *Tet2^–/–^* *Jak2*^V617F^ combined model (14) compared with WT (data not shown). Further, examination of expression changes caused by combined treatment showed a significant reversal of expression patterns induced by *Jak2/IDH* mutations (*P* < 1 × 10^–4^ for both down- and upregulated genes) (Figure 5C). A subset of gene expression signatures reversed by combination therapy were also reversed in mice treated with AG221 monotherapy, and this included both upregulated (*P* < 1 × 10^–4^; NES = +1.9) and downregulated (*P* < 1 × 10^–4^; NES = –2.3) genes. The combined treatment signature showed significant enrichment of gene signatures that were also significantly altered by ruxolitinib monotherapy (downregulated only, *P* < 1 × 10^–4^, NES = –2.2), indicating an additive effect of combined treatment on expression changes relative to that induced by each monotherapy.

We further characterized the differentially expressed gene sets between each treatment group and WT bone marrow. Mice treated with ruxolitinib monotherapy showed loss of enrichment of JAK/STAT-related gene sets compared with vehicle-treated mice. In the case of IL-6/JAK/STAT3, enrichment for the expression of genes in this pathway was seen in vehicle-treated mice and in stem cells from mice treated with AG221 monotherapy. However, this gene expression signature was normalized in stem cells from mice treated with ruxolitinib monotherapy or combined therapy. Similarly, IFN-γ signatures, which were significantly enriched in stem cells from mice treated with vehicle or AG221 monotherapy, lost significance in animals receiving ruxolitinib monotherapy and were negatively enriched in mice treated with combination therapy (Figure 5D). In the context of these findings, we identified several classical oncogene-related gene sets whose gene expression signatures were upregulated in diseased mice and reduced or reversed in treated mice, including signatures derived from cMYC, mTOR, and KRAS. In each of these oncogenic signatures, significant enrichment was present in stem cells from mice treated with vehicle, AG221 monotherapy, and ruxolitinib monotherapy, however, these aberrant gene expression signatures were normalized by or lost statistical significance with combined JAK2/IDH2 inhibitor therapy (Figure 5E). Together, these data indicate that combined treatment restores a WT gene expression pattern in LSK cells.

Given the important role of GATA transcriptional regulators in myelofibrosis pathogenesis (31) and in *Tet2*-mutant AML (15), we performed quantitative PCR for *Gata1* and *Gata2* in donor-derived MEPs isolated from vehicle- and inhibitor-treated mice. Double-mutant recipients treated with vehicle showed a statistically significant increase in *Gata1* expression compared with expression in WT controls (3.242-fold elevation; *P* = 0.0200) (Figure 5F), and this increased expression normalized with single and combined inhibitor treatment (32). *Gata2* levels showed a trend toward increased expression in mice treated with IDH inhibition (Figure 5F), consistent with results in Tet2-mutant AML (15).

### Combined JAK2/IDH2 inhibition has cooperative effects to reverse altered metabolism in JAK2/IDH2-mutant MPN.

**Figure 6:**
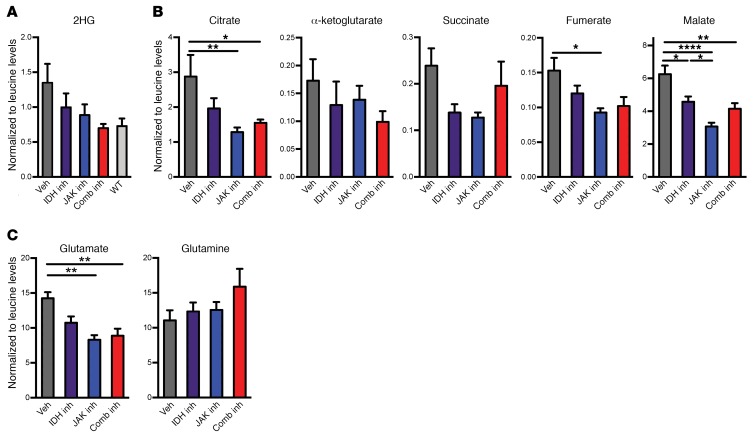
Treatment of combined mutant bone marrow with combined inhibitors perturbs pools of metabolic products. (**A**–**C**) MS analysis of metabolites from whole–bone marrow aspirate cells from treated and WT mice, normalized to leucine levels. (**A**) 2HG levels, (**B**) citrate, α-ketoglutarate, succinate, fumarate, and malate levels, and (**C**) glutamate and glutamine levels. Multiple comparisons were performed using an ordinary 1-way ANOVA with Tukey’s correction for post-hoc comparisons and multiplicity-corrected *P* values. *P < 0.05, **P < 0.01 and ****P < 0.0001.

We next investigated the effect of these treatment regimens on JAK2/IDH2-mutant MPN cell metabolism. We used liquid chromatography/mass spectrometry (LC/MS) to measure metabolites from murine bone marrow aspirates after 10 days of treatment with vehicle, ruxolitinib or AG221 monotherapy, or combined therapy. We first verified that 2HG levels were reduced with IDH inhibitor monotherapy (Figure 6A). IDH inhibitor monotherapy also reduced levels of Krebs cycle intermediates including α-ketoglutarate, citrate, succinate, fumarate, and malate (*P* < 0.0252; Figure 6B). 2HG levels were also reduced with ruxolitinib inhibitor monotherapy (Figure 6A), which is consistent with what we detected in the serum of treated mice (Figure 3A). We also found that ruxolitinib monotherapy reduced pool sizes of citrate (*P* = 0.0064), fumarate (*P* = 0.0224), and malate (*P* < 0.0001; Figure 4G). Consistent with our observations in serum, mice treated with combined therapy had 2HG levels similar to those of WT mice (Figure 6A), indicating a combined effect of the 2 drugs. We also observed a reduction of glutamate levels with ruxolitinib monotherapy (*P* = 0.0026) and combined therapy (*P* = 0.0049) that was accompanied by a coordinate increase in glutamine, particularly in mice that received combination therapy (Figure 6C). These data provide a potential point of intersection between the JAK2- and IDH2-mutant pathways in the regulation of glutamate and glutamine metabolism.

### Inhibition of JAK and mutant IDH shows cooperative efficacy in primary MPN samples from patients with JAK2/IDH2 mutations.

**Figure 7:**
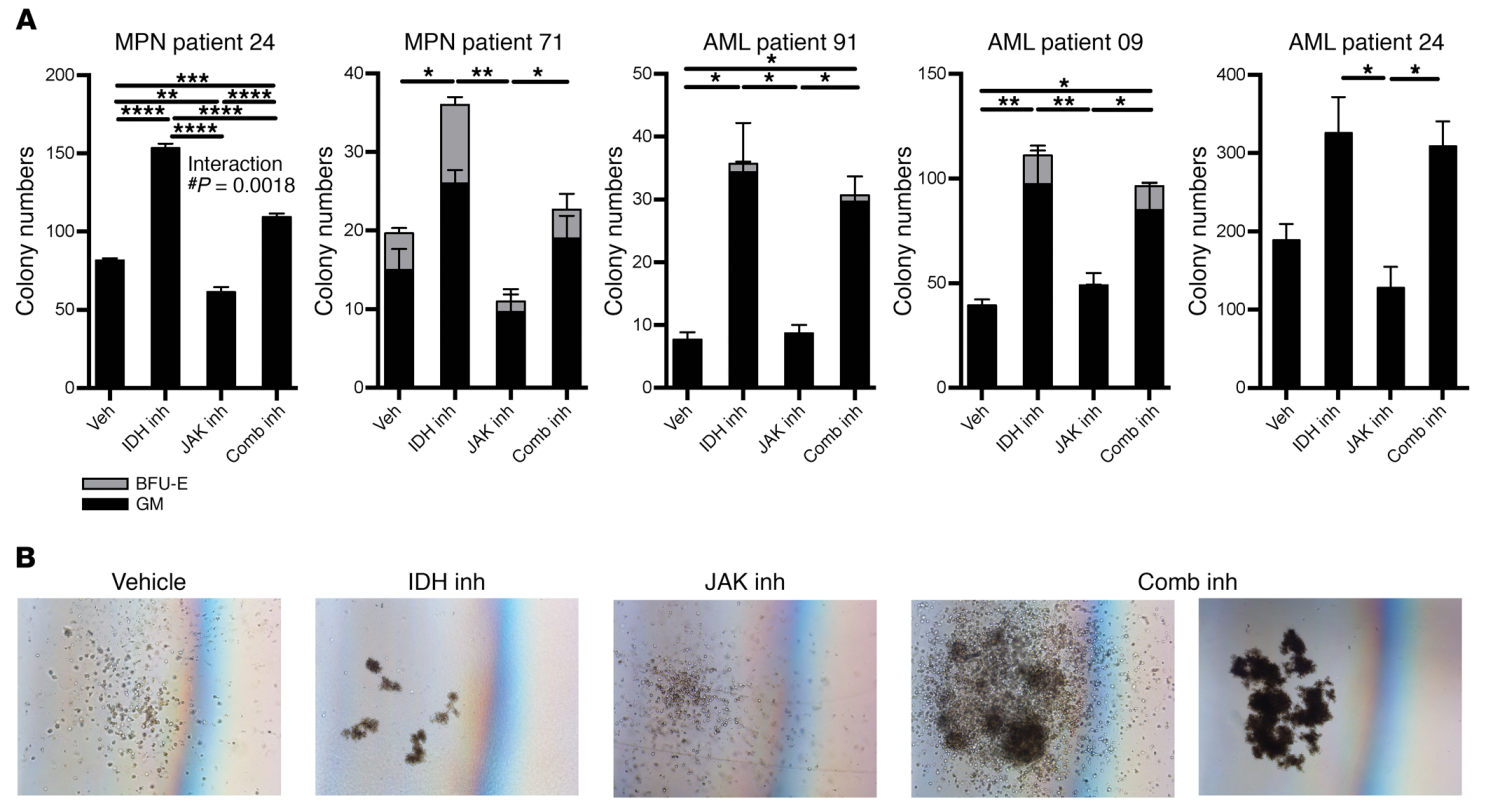
Human *IDH2^R140Q^ JAK2^V617F^* MPN and AML samples in methylcellulose respond to IDH inhibitor therapy with a differentiation phenotype by morphology. (**A**) Colony counts of cultured cells classified by colony morphology including GM and BFU-E colonies. Multiple comparisons were performed using an ordinary 1-way ANOVA, using Tukey’s correction for post-hoc comparisons and multiplicity-corrected *P* values. *P < 0.05, **P < 0.01, ***P < 0.001, and ****P < 0.0001 by 2-way ANOVA. (**B**) Representative images of colonies taken during culturing of cells from MPN patient 71. Magnification ×4. Expression levels of cell-surface markers on cultured cells after therapy as measured by mean fluorescence intensity (MFI) using FACS antibodies: (**C**) CD117, (**D**) CD235a, (**E**) CD14.

**Figure 8:**
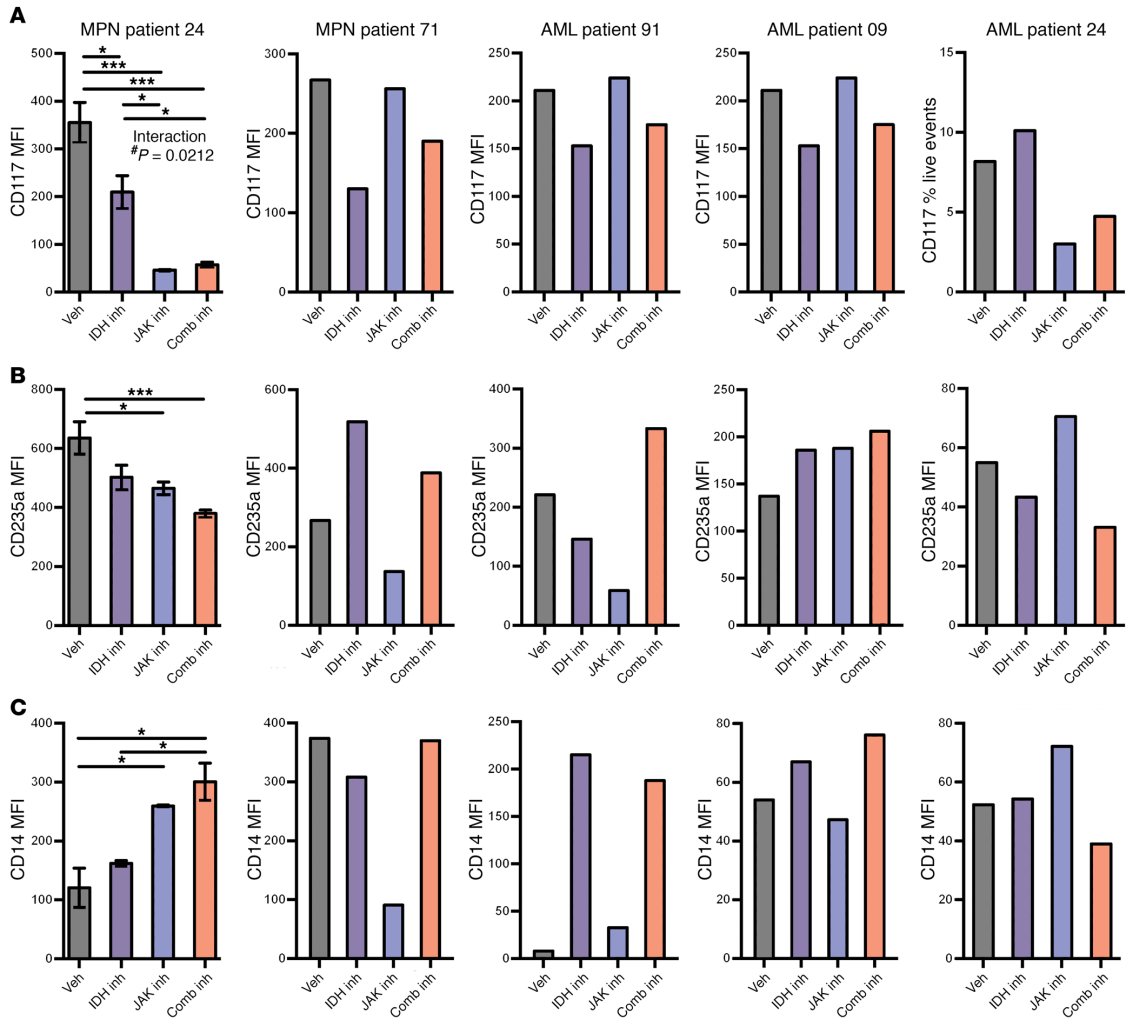
Human *IDH2^R140Q^ JAK2^V617F^* MPN and AML samples in methylcellulose respond to IDH inhibitor therapy with a differentiation phenotype by cell-surface marker measurement. (**A**–**C**) Expression levels of cell-surface markers on cultured cells after therapy as measured by mean fluorescence intensity (MFI) using the following FACS antibodies: (**A**) CD117, (**B**) CD235a, and (**C**) CD14. Multiple comparisons were performed using an ordinary 1-way ANOVA with Tukey’s correction for post-hoc comparisons and multiplicity-corrected *P* values. *P < 0.05, **P < 0.01, and ***P < 0.001. *P* value in **A** obtained by 2-way ANOVA.

To explore the relevance of these findings to human disease, we performed methylcellulose assays on CD34^+^-enriched blood samples from patients with clinically determined MPN and post-MPN AML with *IDH2*^R140Q^ and *JAK2*^V617F^ mutations (Table 1). This analysis included 1 patient for whom combined mutant samples were available from both the chronic-phase MPN and the time of leukemic transformation (patient 24; Figure 7A). All the patients showed a characteristic pattern of colony formation, with an increase in colony numbers with IDH inhibitor monotherapy. The presence of erythroid burst-forming unit (BFU-E) colonies was consistent with the restoration of erythroid differentiation as previously described (25), and 3 of the 5 patients also showed IDH2 inhibition–associated increases in GM colonies, indicating that myeloid differentiation was restored by IDH2 inhibition (Figure 7A). On morphological examination, we found that this expansion was also associated with the presence of large, well-differentiated colonies compared with the control colonies (Figure 7B). FACS analysis showed that IDH2 inhibitor treatment reduced the surface expression of the immature marker c-Kit (also known as CD117) (Figure 8A). With respect to differentiation markers, we found that most patients’ samples treated with AG221 showed upregulation of either the erythroid marker CD235a (Figure 8B) or the myeloid marker CD14, consistent with restored differentiation in JAK2/IDH2-mutant MPN and AML cells (Figure 8C). In contrast, we observed that JAK inhibitor therapy, alone or in combination with IDH2 inhibitor therapy, reduced colony output. Combination treatment attenuated the increase in colony numbers seen with IDH2 inhibitor monotherapy, while still maintaining the effect of IDH2 inhibition on promoting differentiation, as evidenced by a reduction in the proportion of c-Kit^+^ colonies and an increase in the proportion of CD235a^+^ and CD14^+^ colonies, which was not seen with ruxolitinib treatment alone. These data suggest that, while JAK2 inhibitor therapy attenuates proliferation, IDH inhibitor therapy increases cell numbers by promoting differentiation. Therefore, combined JAK and IDH2 inhibitor therapy can have cooperative effects through these 2 responses (33).

## Discussion

Past studies have underscored the importance of mutations in epigenetic regulators in the pathogenesis of myeloid malignancies (34, 35). This includes the neomorphic mutations in IDH1/2 seen in AML and MPN, which, through the production of 2HG, can alter the function of DNA and histone-modifying enzymes (36). Of importance in MPN are the observations that mutations in epigenetic regulators are associated with adverse clinical outcomes and the specific role of IDH1/2 mutations in promoting MPN progression and transformation (9–12, 14, 24, 34, 35). Here, we show that *JAK2* and *IDH* concurrent mutations can promote MPN progression and that this disease is sensitive to combined JAK2/IDH-targeted therapy.

Expression of *Jak2*^V617F^ in concert with *Idh2*^R140Q^ or *IDH1*^R132H^ in hematopoietic cells resulted in an accelerated MPN phenotype with preleukemic features. This disease is characterized by polycythemia and leukocytosis, which were also observed in *Jak2*^V617F^ mice (13). However, expression of mutant IDH1 or IDH2 in concert with *Jak2*^V617F^ resulted in the expansion of blasts and impaired hematopoietic differentiation, which are features of human MPN indicative of disease progression and adverse clinical outcome. These features suggest that expression of IDH1 or IDH2 in concert with *Jak2*^V617F^ impairs differentiation and probably contributes to leukemic transformation, which is consistent with correlative genetic studies showing that the primary effect of IDH1/2 mutations in MPN is that of promoting accelerated progression to post-MPN AML. The phenotype we observed here shares some similarities with the phenotype reported in *Jak2*^V617F^ *Tet2*^null^ mice (14) and is consistent with the observation that mutant IDH–mediated production of 2HG inhibits TET2 catalytic function (19). However, although concurrent *JAK2* and *IDH/TET2* mutations have been reported with high frequency in post-MPN AML (9), the lack of overt acute transformation in mice with these concurrent mutations suggests that there are additional genetic and epigenetic events that are required to induce the transformation from MPN to AML. Importantly, these data are distinct from those of recent work showing that mutant *Idh2/Tet2* can cooperate with *Flt3^ITD^* to potently induce a fully penetrant AML phenotype, suggesting that there are important differences in how different signaling mutations cooperate with epigenetic regulator mutations to induce hematopoietic transformation.

Consistent with this hypothesis, we demonstrate that the MPN stem cell capable of propagating JAK2/IDH-mutant disease is found in the LT-HSC compartment, as observed in *Jak2^V617F^*-driven MPN. By contrast, combined *Flt3^ITD^* and *Tet2*/*Idh2*-mutant leukemia was found to be maintained and propagated by the MPP lineage–restricted progenitor population (15, 16). This could be due to the observation that *JAK2*/IDH-mutant disease remains an accelerated-phase MPN versus the AML observed in combined *Flt3^ITD^*- and *Tet2*/*Idh2*-mutant disease, such that the leukemic stem cell remains in the LT-HSC compartment in chronic-phase disease until blast transformation. Alternatively, it is possible that the expression of different signaling effectors, such as Jak2 or Flt3, differs in different stem/progenitor cell compartments, such that oncogenic signaling induced by different kinase alleles promotes transformation in different cell types, which then dictates the phenotype in concert with mutated epigenetic regulators. Subsequent studies, in which different MPN and AML disease alleles are expressed in specific stem/progenitor cell populations, alone and in concert, are needed to delineate the role of the specific mutations and of the specific cellular compartments in which mutations are acquired in dictating leukemic phenotype and stem cell capacity, because these aspects of hematopoietic disease strongly affect treatment targeting strategies and effects.

Importantly, we demonstrate that *Jak2*/IDH-mutant MPN is highly sensitive to IDH-mutant inhibition and that combined *Jak2*/IDH-mutant–directed targeted therapy has potent antileukemic effects. Specifically, we found that treatment with the IDH2 inhibitor AG221 showed efficacy in *Jak2/Idh2*-mutant MPN, including attenuation of myeloid expansion, reversal of stem cell expansion, and a reduction in the mutant allele burden. Combination therapy with AG221 and ruxolitinib showed increased efficacy without additive toxicity, with reduced disease burden and reversal of aberrant myeloproliferation in vivo. These data provide a rationale for combined JAK2/IDH2-targeted inhibition in this high-risk MPN subtype. Additionally, the apparent suboptimal response of double-mutant mice to a JAK2 inhibitor regimen and the apparent response of double-mutant 2HG levels in serum to single-agent therapy imply a metabolic and gene-regulatory cooperativity between the JAK/STAT pathway and the mutant IDH–mediated effect.

Consistent with the effects on *Jak2/Idh2*-mutant–driven MPN in vivo, combination therapy reversed the aberrant transcriptional and metabolic abnormalities in JAK2/IDH2-mutant stem/progenitor cells. Given the role of mutant IDH enzymes in altering the epigenetic state and gene regulation, it is not surprising that inhibition of mutant IDH function would have a significant impact on gene expression in *Idh2*-mutant stem cells. However, the observation that dual inhibition of JAK signaling and mutant IDH2 function leads to further normalization of aberrant gene expression in JAK2/IDH2-mutant stem/progenitor cells suggests that there is interplay between JAK signaling and epigenetic regulation in driving aberrant gene expression in *Jak2/Idh2*-mutant MPN. *JAK2^V617F^* has been implicated directly in epigenetic regulation of histone modifications (37), but the fact that JAK2 inhibition alone lowered 2HG levels and affected metabolite levels in this study raises the possibility that reversing the effect of JAK signaling on metabolism may influence epigenetic regulation of gene expression. This is plausible, as levels of metabolites other than 2HG can influence the methylation status of DNA and histones, both within the Krebs cycle (38) and in metabolic species beyond it (39). Future studies are needed to delineate specific mechanisms by which constitutive JAK/STAT signaling cooperates with mutant IDH to alter the epigenetic and transcriptional state in hematopoietic cells, but combined inhibition of JAK2 and IDH2 had a greater impact on reversing aberrant metabolism in *Jak2/Idh2*-mutant cells than could be attributed to inhibition of neomorphic IDH2 function alone.

Relevant to patients, the present data suggest an important role for neomorphic IDH mutations in promoting disease progression in concert with activated JAK2 signaling and inform a mechanism-based combination therapy approach for this high-risk MPN subtype. The results of our in vitro experiments using patients’ samples indicate that IDH inhibition expands the population of differentiated cells, which is consistent with the findings in current trials of the drug in de novo AML (33). Given that MPN patients with JAK2/IDH concurrent mutations have a worse prognosis than do patients with other MPN subtypes, there is a need for new therapeutic options. These data provide a rationale for combined, mutant-specific, targeted therapy in this high-risk MPN subtype.

## Methods

### Transgenic animals.

The conditional *Jak2*^V617F^ mice were previously described (13). Conditional transgenic *IDH1*^R132H^ mice (expressing a mutation in the *IDH1* gene) were provided by Kwok-Kin Wong (Dana-Farber Cancer Institute), and conditional *Idh2*^R140Q^ mice were provided by Craig B. Thompson (Memorial Sloan Kettering Cancer Center). All mice were maintained on a C57BL/6J background, and the lines were backcrossed for 6 generations before use in experiments. Samples for experiments were obtained from male and female mice aged 6 months to 1.5 years. Upon induction, mice received 5 i.p. injections of polyI:polyC (Amersham) of 200 μl of a 1-mg/ml solution. Peripheral blood was collected via cheek bleeding using heparinized microhematocrit capillary tubes (Thermo Fisher Scientific). Peripheral blood counts were obtained using a HemaVet according to the manufacturer’s instructions. Excision 2 weeks after induction was confirmed using PCR.

### Histology.

Pathology was obtained after fixation in 4% paraformaldehyde (PFA); blood smears and bone marrow cytospins were performed on the day of sacrifice. Sections were stained with H&E or Wright Giemsa as appropriate. CD34^+^ staining was performed using CD34 rat monoclonal antibody on a Leica Bond RX using the Bond Polymer Refine Detection Kit (catalog DS9800; Leica). The sections stained with CD34 (catalog Ab8158; Abcam; diluted 1:50) were pretreated using heat-mediated antigen retrieval with citrate, pH6 (catalog AR9961; Leica Biosystem Epitope Retrieval 1) for 20 minutes. DAB was used as the chromogen. Samples were further counterstained with hematoxylin and mounted.

### Bone marrow transplantation studies.

Dissected femurs and tibiae were isolated. Bone marrow was flushed into PBS plus 2% BSA or RPMI plus 10% FCS using a syringe or by centrifugal spinning. Spleens were isolated, and single-cell suspensions were made by mechanical disruption using glass slides. All harvested cells were passed through a 70-mm strainer. RBC were lysed in ammonium chloride–potassium bicarbonate lysis buffer for 10 minutes on ice. Cells were transplanted via tail-vein injection into lethally irradiated (2 × 5.50 Gy) CD45.1 host mice (The Jackson Laboratory). For noncompetitive transplants, 1 × 10^6^ total cells were transplanted; for competitive transplants including drug studies, 1 × 10^6^ total donor cells were injected into a mixture with 1 × 10^6^ cells from a congenic CD45.1 donor; for cell-of-origin transplants, the injection number was determined on basis of the lowest yield after sorting to inject approximately 100,000 MPPs or 300 LT-HSCs with 300,000 whole bone marrow cells from a CD45.1 donor.

### Therapeutic assays.

Approximately 2 months after competitive transplantation, peripheral blood analysis was performed with a HemaVet and FACS for donor chimerism. Mice were matched using *Z* scores representing hematocrit (HCT) (percentage), WBC (K/μl), donor chimerism, and body weight, and mice were randomized within matching groups using a random number generator. Drugs were administered twice daily by gavage for 21 to 28 days, unless otherwise indicated. Ruxolitinib (a gift of James Bradner, Dana-Farber Cancer Institute) was administered at a dose of 60 mg/kg, and AG221 (Agios Pharmaceuticals) was administered at a dose of 100 mg/kg or 40 mg/kg. Final doses were administered approximately 1.5 hours before sacrifice.

### Flow cytometry and FACS for murine tissues.

Cells were stained with antibodies in FACS buffer (2% BSA in PBS) for 30 minutes on ice. Donor and support chimerism was assessed using antibodies against CD45.1 (clone A20; BioLegend) and CD45.2 (clone 104; BioLegend).

For hematopoietic stem and progenitor cell staining, cells were stained with a lineage cocktail consisting of CD4 (clone RM4-5; BioLegend), CD3 (clone 17A2; BioLegend), B220/CD45R (clone RA3-6B2; BioLegend), NK1.1 (clone PK136; BioLegend), Gr-1 (clone RB6-8C5; BioLegend), Mac1/CD11b (clone M1/70; BioLegend), and Ter119 (catalog 116223; BioLegend) and allowing for mature lineage exclusion from the analysis. Cells were also stained with antibodies specific to c-Kit/CD117 (clone 2B8; BioLegend), Sca-1 (clone D7; BioLegend), FcγRII/III/CD16/32 (clone 2.4G2; eBiosciences), and CD34 (clone RAM34; eBiosciences) or SLAM/CD150 (clone TC15-12F12.2; BioLegend) and CD48 (clone HM48-1; eBioscience). To assess mature cell lineages, a combination of antibodies against Mac1, Gr-1, B220, CD3, c-Kit/CD117, CD45.1, and CD45.2 was used.

To assess erythroid and megakaryocyte progenitors, we stained unlysed tissues with a lineage cocktail consisting of CD4, CD8, B220/CD45R, Mac1/CD11b, Gr-1, IL7R/CD127 (clone A7R34; BioLegend), and CD49b (clone DX5; BioLegend). Antibodies against c-Kit/CD117, Sca-1, SLAM/CD150, CD48, FcγRII/III/CD16/32, CD41 (clone eBioMWReg30; eBioscience), CD105 (clone MJ7/18; BioLegend), CD71 (clone RI7217; BioLegend), and Ter119 were also used and gated as previously described (40).

Before cell-of-origin transplantations, sorting was performed on bone marrow with enrichment for c-Kit^+^ cells using CD117 MicroBeads (Miltenyi Biotec). For expression analysis, sorting was performed on bone marrow that had been depleted of mature cells using a Progenitor Cell Enrichment Kit (STEMCELL Technologies).

### Metabolomic analysis.

Analysis of serum 2HG was performed by extraction with 80% aqueous methanol as previously described (41). All extracts were spun at 13,000 rpm at 4°C to remove precipitate, dried at room temperature, and stored at –80°C. Metabolite levels were determined by ion-paired, reverse-phase LC coupled to negative-mode, electrospray triple-quadrupole MS using multiple reactions monitoring, and integrated elution peaks were compared with metabolite standard curves for absolute quantification (17).

For central carbon metabolite profiling of flash-frozen bone marrow aspirates, polar metabolites were extracted from flash-frozen bone marrow aspirates using 5:3:5 ice-cold methanol/water/chloroform on ice. Extracts were vortexed for 10 minutes at 4°C and then centrifuged for 5 minutes at 4°C at maximum speed on a tabletop centrifuge. Equal volumes of the aqueous phase from each sample were removed and dried under nitrogen gas and then resuspended for LC/MS analysis, and 1 μl of each sample was injected for hydrophilic interaction–LC/MS (HILIC-MS analysis) on a Dionex Ultimate 3000 UPLC System coupled to a QExactive Orbitrap Mass Spectrometer as previously described (42).

### Expression analysis.

CD45.2^+^ LSK cells were sorted into ice-cold FACS buffer, pelleted, resuspended, and stored in TRIzol (Invitrogen/Thermo Fisher Scientific) until extraction of RNA using phenol-chlorophorm. The library was produced using SMARTer amplification (Clontech Laboratories) to amplify and create the library. Illumina sequencing was performed using paired-end 50 bp at 40 × 10^6^ reads per sample.

FASTQ files were aligned to mm10 using STAR, version 2.5.1a (43) with default parameters. Counts were generated using the Ensembl gene model (https://www.ensembl.org/info/genome/genebuild/genome_annotation.html) and STAR, version 2.5.1a, with the —quantMode parameter. Differential expression analysis was performed using the Bioconductor DESeq2 package (44) with default parameters. Heatmaps were generated using the CRAN NMF package (45) and normalized counts as provided by DESeq2. Gene set enrichment analysis (GSEA) (46, 47) was performed using MSigDB Hallmark gene sets (http://software.broadinstitute.org/gsea/msigdb/collections.jsp). Custom gene sets for JAK2/IDH disease were generated using all genes that were significantly differentially expressed, with Benjamini-Hochberg–adjusted *P* values of less than 0.0001.

### Quantitative PCR for mRNA.

CD45.2^+^ MEPs were sorted into ice-cold FACS buffer, pelleted, resuspended, and stored in TRIzol until extraction of RNA using phenol-chloroform. The RNA was then used as a template for cDNA synthesis using the Verso cDNA Kit (Thermo Fisher Scientific). Quantitative real-time PCR reactions were carried out using SYBR Green Master Mix (Life Tech) on an Applied Biosciences qPCR cycler. Relative expression was determined by the Δ/ΔCt method and normalized to the internal control GAPDH.

### Human tissues.

Fresh peripheral blood was collected into heparinized collection tubes, and separation of peripheral blood mononuclear cells (PBMCs) was performed using hetastarch and a Ficoll gradient with subsequent RBC lysis.

### Human colony-forming assays.

Frozen or fresh PBMCs were plated in MethoCult H4435 (STEMCELL Technologies) with penicillin and streptomycin in triplicate wells. Cells from MPN patients were plated after enrichment using CD34 Microbeads (Miltenyi Biotec) at 1,000 cells per well, and cells from AML patients were plated without enrichment at 100,000 cells per well. AG221 and ruxolitinib were dissolved into samples at a 400-nM concentration, and DMSO was dissolved into the controls.

### Flow cytometry for human tissues.

To observe erythroid differentiation in human tissues, staining was performed with a combination of CD117/c-Kit (clone YB5.B8; eBioscience), CD34 (clone 581; BioLegend), CD38 (clone HIT2; BioLegend), CD36 (clone 5-271; BioLegend), CD71 (clone OKT9; eBioscience), and CD235a (clone HIR2; eBioscience). To observe monocytic and granulocytic differentiation, antibodies against CD117/c-Kit, CD34, CD38, CD15 (clone HI98; eBioscience), CD14 (clone HCD14; BioLegend), and CD16 (clone 3G8; BioLegend) were used.

### Statistics.

Data are presented as the mean ± SEM. GraphPad Prism 7 software (GraphPad Software) was used to conduct the statistical analysis of all data. Multiple comparisons were performed using an ordinary 1-way ANOVA with Tukey’s correction for post-hoc comparisons and multiplicity-corrected *P* values. Survival comparisons were made using the log-rank (Mantel-Cox) test. Statistical interaction calculated for combined influence of JAK and IDH mutation or inhibition status combined was determined using 2-way ANOVA. Paired *t* tests were used to determine 2-tailed significance to compare results in mice before and after treatment. A *P* value of less than 0.05 was considered statistically significant.

### Study approval.

All animal procedures were conducted in accordance with the NIH guidelines for the care and use of laboratory animals and approved by the IACUC of Memorial Sloan Kettering Cancer Center. Tissue samples were collected in partnership with the Human Oncology Tissue Bank, and all patients provided written informed consent. Approval for the use of human samples was obtained from the IRB of Memorial Sloan-Kettering Cancer Center.

### Data and materials availability.

RNA-seq data used in this work were deposited in the NCBI’s Gene Expression Omnibus (GEO) database (GEO GSE95771).

## Author contributions

ASM, ANL, MGVH, CBT, and RLL designed the experiments. ASM, ANL, and RLL wrote the manuscript. All authors edited the manuscript. AC and JTF performed analysis of histological sections. ANL, AMI, EF, and MS performed MS analysis on primary samples. BS and FTR ran the computational pipelines. AMI, AHS, EAA, and KW designed the backcrossing of the transgenic mice for experimentation. ASM, AVHS, JA, KS, and EP performed mouse husbandry, transgenic mouse experiments, and therapeutic gavage experiments. RN and KY provided advice on the use of AG221. ASM, AVHS, JA, MAP, and RR identified, collected, and aided in the analysis of human samples.

## Supplementary Material

Supplemental data

## Figures and Tables

**Table 1:**
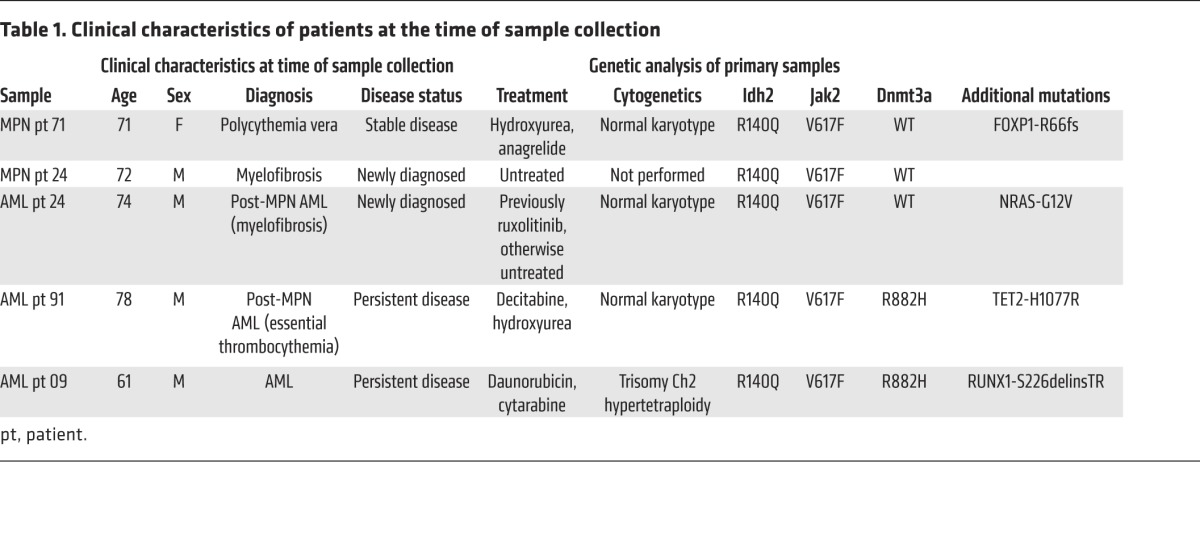
Clinical characteristics of patients at the time of sample collection

## Version 1 01/22/2018

Electronic publication

## Version 2 02/01/2018

Print issue publication
