# Supplementary material for: JAK2/IDH-mutant–driven myeloproliferative neoplasm is sensitive to combined targeted inhibition
Source: J Clin Invest. 2018 Jan 22;128(2):789–804. doi: 10.1172/JCI94516 (PMC5785272; doi:10.1172/JCI94516)
Supplement: Supplemental data [file jci-128-94516-s001.pdf]

**Supplementary Materials:**

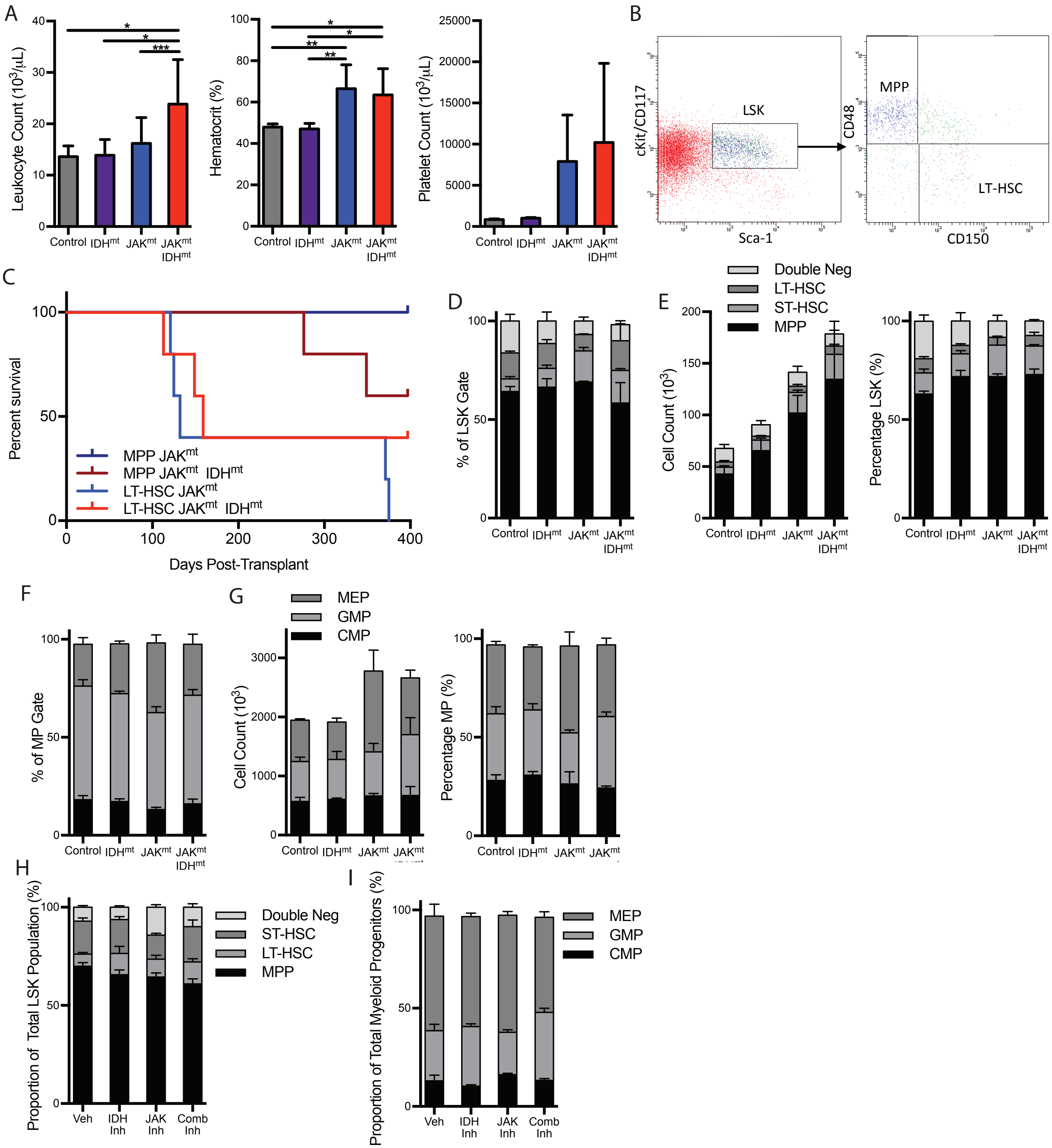

A
Leukocyte Count (10^3/µL)
Hematocrit (%)
Platelet Count (10^3/µL)
Control
IDHmt
JAKmt
JAKmt IDHmt
B
cKit/CD117
Sca-1
LSK
CD48
CD150
MPP
LT-HSC
C
Percent survival
Days Post-Transplant
MPP JAKmt
MPP JAKmt IDHmt
LT-HSC JAKmt
LT-HSC JAKmt IDHmt
D
% of LSK Gate
E
Double Neg
LT-HSC
ST-HSC
MPP
Cell Count (10^3)
Percentage LSK (%)
F
% of MP Gate
G
MEP
GMP
CMP
Cell Count (10^3)
Percentage MP (%)
H
Proportion of Total LSK Population (%)
Double Neg
ST-HSC
LT-HSC
MPP
Veh
IDH Inh
JAK Inh
Comb Inh
I
Proportion of Total Myeloid Progenitors (%)
MEP
GMP
CMP

**Supplemental Figure 1: Combined mutant mouse phenotype A,** Peripheral blood leukocyte counts and hematocrit from recipients of competitive transplants 12 weeks post-transplant. **B,** Representative image of gates used to sort SK populations for injection into recipients. **C,** Survival curve of mice injected with different LSK populations **D,** LSK populations in primary *Idh2*$^{R140Q}$ *Jak2*$^{V617F}$ mice represented as proportions of total LSK. **E,** LSK populations in primary *IDH1*$^{R140Q}$ *Jak2*$^{V617F}$ mice represented as totals and proportions of total LSK. **F,** Myeloprogenitor populations in primary *Idh2*$^{R140Q}$ *Jak2*$^{V617F}$ mice represented as proportions of total LSK. **G,** Myeloprogenitor populations in primary *IDH1*$^{R140Q}$ *Jak2*$^{V617F}$ mice represented as totals and proportions of total LSK. **H,** LSK, and **I,** Myeloprogenitor compartments expressed as proportions of subcompartments in *Idh2*$^{R140Q}$ *Jak2*$^{V617F}$ bone marrow recipients treated with targeted inhibitors. *P<0.05, **P<0.01, ***P<0.001, ****P<0.0001.
